# Molecular Crosstalk Between Macrophages and Mesenchymal Stromal Cells

**DOI:** 10.3389/fcell.2020.600160

**Published:** 2020-12-09

**Authors:** Hazel Y. Stevens, Annie C. Bowles, Carolyn Yeago, Krishnendu Roy

**Affiliations:** ^1^Marcus Center for Therapeutic Cell Characterization and Manufacturing, Parker H. Petit Institute for Bioengineering and Bioscience, Georgia Institute of Technology, Atlanta, GA, United States; ^2^NSF Engineering Research Center (ERC) for Cell Manufacturing Technologies (CMaT), Georgia Institute of Technology, Atlanta, GA, United States; ^3^The Wallace H. Coulter Department of Biomedical Engineering, Georgia Institute of Technology, Emory University, Atlanta, GA, United States; ^4^Center for ImmunoEngineering, Georgia Institute of Technology, Atlanta, GA, United States

**Keywords:** macrophages (M1/M2), mechanism of action (MOA), immunomodulation, cell therapy, mesenchymal stromal (or stem) cells

## Abstract

Mesenchymal stromal cells (MSCs) have been widely investigated for regenerative medicine applications, from treating various inflammatory diseases as a cell therapy to generating engineered tissue constructs. Numerous studies have evaluated the potential effects of MSCs following therapeutic administration. By responding to their surrounding microenvironment, MSCs may mediate immunomodulatory effects through various mechanisms that directly (i.e., contact-dependent) or indirectly (i.e., paracrine activity) alter the physiology of endogenous cells in various disease pathologies. More specifically, a pivotal crosstalk between MSCs and tissue-resident macrophages and monocytes (TMφ) has been elucidated using *in vitro* and *in vivo* preclinical studies. An improved understanding of this crosstalk could help elucidate potential mechanisms of action (MOAs) of therapeutically administered MSCs. TMφ, by nature of their remarkable functional plasticity and prevalence within the body, are uniquely positioned as critical modulators of the immune system – not only in maintaining homeostasis but also during pathogenesis. This has prompted further exploration into the cellular and molecular alterations to TMφ mediated by MSCs. *In vitro* assays and *in vivo* preclinical trials have identified key interactions mediated by MSCs that polarize the responses of TMφ from a pro-inflammatory (i.e., classical activation) to a more anti-inflammatory/reparative (i.e., alternative activation) phenotype and function. In this review, we describe physiological and pathological TMφ functions in response to various stimuli and discuss the evidence that suggest specific mechanisms through which MSCs may modulate TMφ phenotypes and functions, including paracrine interactions (e.g., secretome and extracellular vesicles), nanotube-mediated intercellular exchange, bioenergetics, and engulfment by macrophages. Continued efforts to elucidate this pivotal crosstalk may offer an improved understanding of the immunomodulatory capacity of MSCs and inform the development and testing of potential MOAs to support the therapeutic use of MSCs and MSC-derived products in various diseases.

## Introduction

Mesenchymal stromal cells (MSCs), also referred to as Mesenchymal Stem Cells or Medicinal Signaling Cells, have garnered attention as cell therapies against various diseases, including graft versus host disease (GvHD) (Ball et al., 2013), neurological disorders (Riordan et al., 2019; Sun et al., 2020), and cardiovascular disease (Hare et al., 2009). For decades, MSCs have been investigated for their immunomodulatory, anti-inflammatory, and regenerative functions revealing several potential modalities for mediating therapeutic effects. A large body of literature has demonstrated that MSCs are highly responsive to environmental cues and elicit their effects through direct [i.e., cell–cell contact (Quaedackers et al., 2009; Galipeau and Sensebe, 2018)] and indirect (i.e., paracrine signaling (Caplan and Correa, 2011; Salgado and Gimble, 2013; Serejo et al., 2019)] interactions resulting in suppression of pathogenic cells (Ren et al., 2008; Quaedackers et al., 2009), induction of regulatory cells (Luz-Crawford et al., 2013; Lee et al., 2017), cytoprotection (Block et al., 2009), trophic support (Zhang et al., 2007), and tissue repair (Wu et al., 2007). Thus, the therapeutic capacity of exogenously administered MSCs relies on their innate ability to respond to surrounding pathophysiological cues and orchestrate cellular and molecular changes to restore local and systemic homeostasis.

Although MSCs hold promise for clinical use, our knowledge of MSCs has been gained primarily through *in vitro* assays and pre-clinical animal studies, leaving gaps in translation and an inability to demonstrate definitive efficacy in human clinical trials (Galipeau and Sensebe, 2018). How we characterize MSCs and test their therapeutic “potencies” *ex vivo* may be the reason for these disparities that are observed between preclinical and clinical trial results. Further elucidations into cellular and molecular interactions mediated by MSCs will better inform future investigations of key endogenous cellular targets and, ultimately, bridge the gaps to advance clinical use of MSCs by understanding how, when, and where to deliver therapeutic MSCs.

MSCs have been isolated from various tissues of the body including bone marrow, adipose, and umbilical cord tissue. By harnessing the plastic-adherence property of MSCs and with the addition of a tailored media formulation for sustaining their growth, the residual tissue-resident cells can be eliminated and MSCs can be obtained for investigational use (Secunda et al., 2015; Palumbo et al., 2018). Culture systems enable expansion of MSCs for acquiring the necessary cell numbers (i.e., yield) for therapeutic dosages in humans or animal studies. Regardless of the tissue source, plastic-adherent MSCs are further characterized by a specific set of criteria such as their expression profile of positive (e.g., CD73, CD90, and CD105) and negative (e.g., CD11b, CD14, CD19, CD34, CD45, CD79a, and HLA-DR) surface markers and *in vitro* multi-lineage differentiation capacity (i.e., induced osteogenesis, adipogenesis, and chondrogenesis) (Dominici et al., 2006). The caveat, however, is that *ex vivo* manipulation to isolate, expand, and interrogate MSCs may introduce transcriptional, epigenetic, metabolomic, and proteomic changes – and these characteristics of cultured MSCs likely do not parallel those of endogenous stromal cells (Caplan, 2008). Moreover, delivery of therapeutic MSCs back to an *in vivo* environment makes it additionally challenging to anticipate outcomes and demonstrate reproducible results. For these reasons, we will primarily focus on MSCs infused as therapy and the consequential effects on endogenous cells.

Autologous or allogeneic MSCs delivered as therapy may exert multiple effects to mitigate local and systemic pathologies. These potential therapeutic modalities are a result of the dynamic ability of MSCs to respond to various stimuli (Caplan and Correa, 2011). The caveat, however, is that these multimodal effects of MSCs make it challenging to identify specific mechanisms of action (MOA) that, if realized, can then be exploited in developed testing platforms. In fact, there is a strong need to develop and test a multivariate set of assays to evaluate mechanistic outcomes of MSCs using co-cultures with immune cells. Not only would understanding biological variation, evaluating manufacturing processes, and evaluating tissue sources be improved, these assays would help predict *in vivo* “therapeutic potencies” of infused MSCs, ultimately facilitating the translation and standardization necessary to advance cell manufacturing and regulatory approval for clinical use (Bianco et al., 2008; Chinnadurai et al., 2018). *In vitro* and *in vivo* preclinical studies have, thus far, provided compelling evidence for key interactions, referred to as molecular crosstalk, between MSCs and immune cells, most notably monocytes, macrophages, and T lymphocytes (Bianco et al., 2008; Chinnadurai et al., 2018; Antebi et al., 2019). These vital immune cells are at the crux of immune system functions, transmitting information in the form of molecular signals from a site of pathology to the rest of the body. Here, we take a more in-depth look into evidence that suggests an integral crosstalk between MSCs and specifically monocytes and tissue-resident macrophages (TMφ) to get us one step closer to identifying potential MOAs by MSCs.

## Changing Dogma Delineate Monocyte and Macrophage Populations

First, understanding the physiological roles of TMφ during steady-state (i.e., homeostasis) and pathology is necessary to realize the alterations mediated by MSCs, and vice versa. Monocytes and macrophages, as well as dendritic cells, comprise the mononuclear phagocytic system (MPS). More in-depth physiological roles will be further described herein, but the most simplified term that captures the functions of MPS cells is “SHIP” – Sample, Heal, Inhibit, and Present (antigen) (Mills et al., 2014). Together, MPS cells are essential cells of the innate immune system that acquire information from their surroundings (e.g., by phagocytosis) and communicate the information (e.g., by antigen-presentation) to the adaptive immune system for a coordinated resolution of pathology. Thus, physiological plasticity (i.e., functional heterogeneity) is the underlying propensity of these cells to regulate tissue microenvironments (Wynn et al., 2013). With the dynamic nature of both MSCs and TMφ, determining the mechanistic effects resulting from this integral crosstalk continues to be an ongoing exploration.

The literature depicting the physiology of macrophages often pays homage to the pioneering work of Ilya Metchnikoff, a Russian zoologist turned immunologist and Nobel Prize laureate. He not only described these “large devouring cells” in the late 19th century but, more importantly, suggested the role of these cells as part of the host’s defenses, the first implications of the innate immune system, deeming him the father of cellular immunity (Hoeffel and Ginhoux, 2015). Discoveries after that showed commonalities in cellular responses and phagocytic functions between bone marrow-derived monocytes and tissue-resident macrophages that led many to believe that monocytes were the predecessors of macrophages. Although these physiological similarities are still appreciated, fate mapping and lineage tracing technologies have more recently delineated their ontogenies. Hematopoiesis in the bone marrow generates myeloid precursors that differentiate into monocytes upon emigration to the bloodstream. TMφ can develop from circulating monocytes that have infiltrated into tissues. However, most of them originate from either yolk sac-derived erythro-myeloid or fetal liver progenitors during embryogenesis, i.e., hematopoietic stem cell-independent precursors (Dey et al., 2014; Hoeffel and Ginhoux, 2015). Further delineation of monocytes and macrophages describes differentiation into subsets and transitional phenotypes, respectively, with distinct functions influenced by spatiotemporal cues.

Monocytes are generally categorized into subsets corresponding to surface markers and functional activities, some of which are shared with TMφ. Interestingly, the frequency of each monocyte subset is inversely related to their lifespan during a steady state. Classical monocytes (CD14^high^CD16^–^) make up about 85% of the circulating monocytes, and 15% consist of both intermediate (CD14^high^CD16^+^) and non-classical monocytes (CD14^low^CD16^high^) (Patel et al., 2017; Ong et al., 2019). Classical monocytes are considered less mature and emerge from the bone marrow, where they enter the circulatory system with a propensity for phagocytosis of debris or foreign invaders, with the shortest lifespan of 1 day. The majority of these cells die or extravasate into tissues, whereas a small portion transition into intermediate monocytes. Intermediate monocytes are generated in response to an initial stimulus and function to propagate inflammatory signaling over a lifespan of a little over 4 days. Intermediate monocytes then transition to non-classical monocytes, with have an extended lifespan of approximately 7.5 days, allowing them to patrol the vasculature and potentially infiltrate affected tissues to resolve the inflammatory stimulus (Thomas et al., 2015; Patel et al., 2017). Thus, intermediate and non-classical monocytes are considered the mature “inflammatory” subsets as their frequency is elevated in the blood during inflammation or pathogenesis (Hamilton and Tak, 2009; Thomas et al., 2015). Although circulating monocytes have been observed to extravasate into tissues, especially during pathological activities, the majority provide short-term surveillance as a host’s first line of defense and are then replenished by continued hematopoiesis (van Furth and Cohn, 1968).

In contrast, tissue-resident macrophages possess dynamic phenotypes and functions, some of which exhibit tissue-specific functions. Here, TMφ self-renew and persist for months to even years in steady-state. Upon activation by inflammation or other pathological stimuli, naïve macrophages (M0) differentiate into classical or alternative activation macrophages, formerly M1 or M2 phenotypes, respectively, according to their surrounding microenvironment. Initially, the M1 or M2 phenotypes denoted a pro- or anti-inflammatory function, respectively, and this paradigm was synonymous with the polarized responses of toll-like receptor (TLR) signaling observed with T lymphocyte subset (i.e., type 1 or 2 helper T cells) (Mills et al., 2000). Although this macrophage nomenclature is still used, emergent evidence now suggests a spectrum of spatiotemporal identifiers, ultimately relinquishing these finite conventions (Nahrendorf and Swirski, 2016). The dynamic phenotypes of the classical or alternative activation macrophage subsets have been identified to capture physiological functions closely correlated to metabolic programs (Mills et al., 2000; Vasandan et al., 2016). A burgeoning research area endeavors to ascertain their spatiotemporal role, however, this review will collectively consider macrophages regardless of the nomenclatures describing subsets, phenotypes, and tissue-specific names to describe their crosstalk with MSCs (Table 1).

**TABLE 1:** Nomenclature used to denote monocyte and macrophage subsets with associated phenotypic markers and functions found in humans.

Nomenclature	Phenotypic markers	Function	Citation
Classical monocytes (Naïve)	HLA-DR^+^, CD11b^+^, CD14^high^, CD16^–^, CCR2^high^, CXC3R1^low^	Phagocytosis	Ong et al., 2019; Thomas et al., 2015
Intermediate monocytes (activated)	HLA-DR^+^, CD11b^+^, CD14^high^, CD16^+^, CCR2^+^, CXC3R1^high^ CCR5^+^	Pro-inflammatory	Thomas et al., 2015; Ong et al., 2019
Non-classical monocytes (activated)	HLA-DR^+^, CD11b^+^, CD14^low^CD16^high^, CCR2^low^, CX3CR1^high^	Patrolling	Thomas et al., 2015; Ong et al., 2019
Classical activation (M1) TMφ	HLA-DR^+^, CD68^+^, CD80^high^, CD206^low^ CD40^+^, CCR7^+^, CXCL9^+^, IL-10^low^, IL-12^high^	Pro-inflammatory, microbicidal, Th1 differentiation, tumor resistance	Martinez et al., 2008; Raggi et al., 2017; Shapouri-Moghaddam et al., 2018
Alternative activation (M2) TMφ	HLA-DR^+^, CD68^+^, CD86^+^, CD80^low^, CD206^high^, CD36^+^	Anti-inflammatory, reparative	Martinez et al., 2008; Raggi et al., 2017; Shapouri-Moghaddam et al., 2018
M2a subset	CD206+, IL-1R^+^, CCL17^+^	Anti-inflammatory, promote cell growth, wound healing	Martinez et al., 2008; Shapouri-Moghaddam et al., 2018
M2b subset	CD86^+^, CCL1^+^, IL-10R^+^, IL-12R^+^, IL-6R^+^, IL-10^high^, IL-12^low^	Immunoregulation, promote tumor progression, Th2 differentiation, microbial infections	Martinez et al., 2008; Shapouri-Moghaddam et al., 2018
M2c subset	CD206^+^, CD163^+^, CXCL13^+^, TLR-1^+^, TLR-8^+^	Immunosuppression, phagocytosis, tissue remodeling	Martinez et al., 2008; Shapouri-Moghaddam et al., 2018
M2d subset	IL-10R, IL-12R, IL-10^high^, IL-12^low^, TNFα^low^	Angiogenesis, tumor progression	Martinez et al., 2008; Shapouri-Moghaddam et al., 2018

*Alternative activation (M2) TMφ are further delineated into four subsets (a–d) with specific markers and functions.*

## Functional Heterogeneity of Macrophages

**FIGURE 1:**
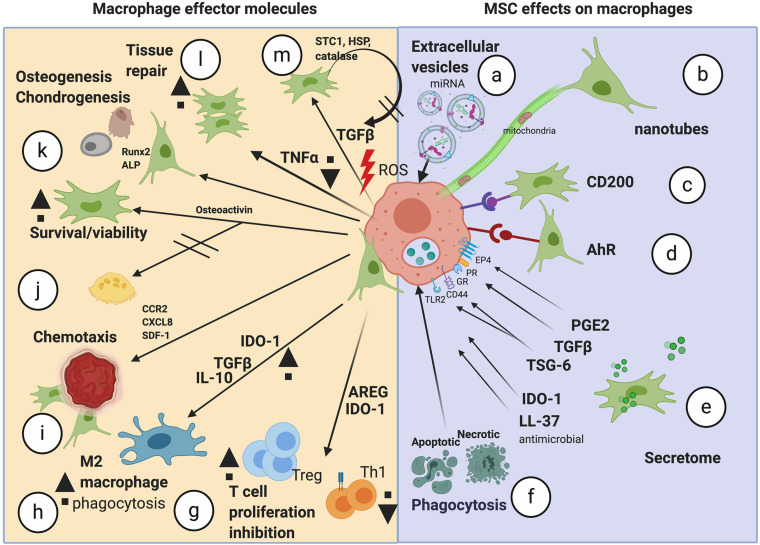
Schematic of the potential mechanisms of action mediated by MSCs on TMφ. The range of mechanisms important in MSC effects on macrophages (a–f) and the resulting effector molecules/effects seen in macrophages (g–m). miRNA, microRNA; AhR, aryl hydrocarbon receptor; PGE2, prostaglandin E2; TGFβ, transforming growth factor beta; TSG-6, TNF alpha-stimulated gene 6; IDO-1, indoleamine 2,3 dioxygenase; LL-37, antimicrobial peptide; AREG, amphiregulin; Tregs, regulatory T cells; IL-10, interleukin-10; CCR2, monocyte chemotactic protein −1 receptor; CXCL8, chemokine C-X-C motif chemokine ligand 8; SDF-1, stromal cell derived factor 1; Runx2, Runt-related transcription factor 2; ALP, alkaline phosphatase; LPS, lipopolysaccharide; TNFα, tumor necrosis factor alpha; ROS, reactive oxygen species; HSP, heat shock protein; STC1, stanniocalcin-1; Image created with BioRender.

TMφ are vital cells of the body that are integral to the development and homeostatic maintenance of all tissues (Figure 1). These professional phagocytes have shared roles and responses across tissues, including clearance of dead cells and debris, presenting antigen, remodeling of tissue, and metabolic regulation but also functions that serve tissue-specific demands related to each organ. Interestingly, some physiological functions enable others. For instance, one of the primary TMφ of the bone, osteoclasts, are responsible for bone resorption which is fundamental to bone remodeling during development; thus, osteoclasts sculpt the bone cavity, enabling hematopoiesis to ensue. Consequently, the generation of monocytes and subsequently immune system functions rely on these bone resident TMφ. Throughout the body, these shared roles act specifically to maintain steady state functions of resident cells and organ system functions.

During erythropoiesis, macrophages surround maturing erythroblasts, ingest extruding nuclei and essentially permit formation of erythrocytes, or red blood cells. The depletion of erythrocytes for natural turnover is also a steady state function of splenic and hepatic TMφ. The liver, pancreas, and adipose tissue are organs that maintain metabolic homeostasis. TMφ of the liver, called Kupffer cells, facilitate the metabolism of hepatocytes during caloric intake, regulating the uptake, synthesis, and oxidation of fatty acids. Similarly, TMφ support β-cell function in the pancreas, although their precise role during steady state remains to be determined since discoveries so far appear to be a consequence of pancreatic dysfunction, such as insulin resistance. Insulin and other hormone sensitivities are maintained by TMφ in adipose tissue for systemic metabolic regulation and thermogenic control of the body (Hamilton and Tak, 2009; Wynn et al., 2013).

Microglia are the main brain-resident TMφ responsible for neuronal patterning, survival, and function while other TMφ in the brain are localized to key areas for maintaining fluid balance and the integrity of the blood-brain barrier (Chen et al., 2015). Understandably, TMφ are fundamental constituents of the heart, lungs, and other organ systems, playing key roles during development and throughout adulthood; however, it is mostly through inflammatory and pathological conditions that their roles are elucidated. It is of no surprise that TMφ are involved in almost every disease and crosstalk with neighboring stromal cells serves as a vital connection to restore homeostasis of tissues.

## Cellular Crosstalk With MSCs

**FIGURE 2:**
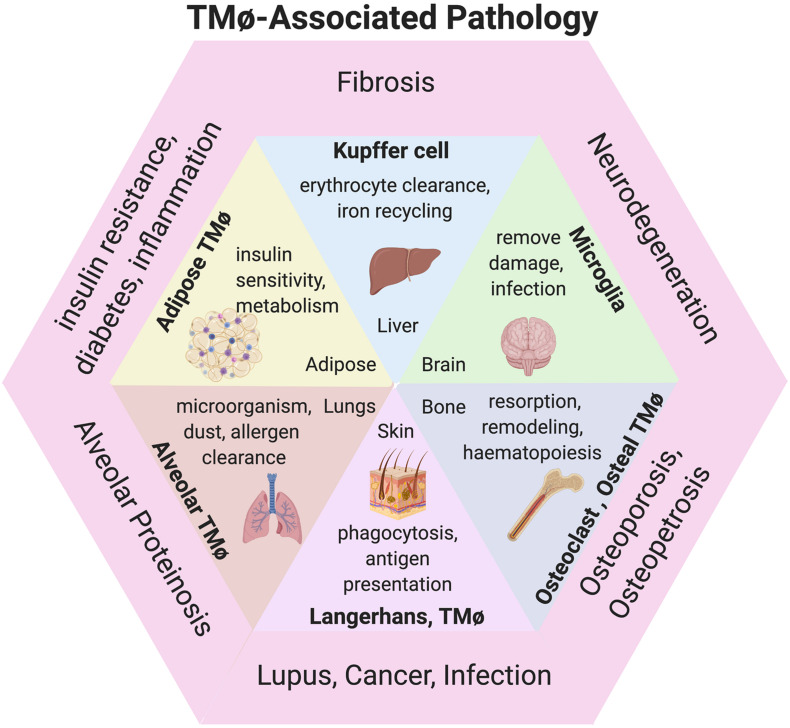
TMφ-associated pathologies. Diagram of TMφ functions described during physiological (inside) and pathological presentations of disease (outer) corresponding to organs. Image created with BioRender.

Several of the proposed MOAs that have been implicated as the underlying therapeutic effects of administered MSCs directly or indirectly acts on TMφ (Figure 2). Initial pathogenesis or physical insult mount a “SOS” response, recruiting cells (e.g., TMφ) that are proximal and distal to the site of pathology by chemotactic and tropic factors. MSCs, too, have demonstrated an ability to home to sites of pathology once infused as therapy (Shi et al., 2007). However, the most common route of delivery for MSCs is currently by intravenous infusion, rendering MSCs lodged in the capillaries of the lungs soon after and no evidence of engraftment to date (Giri and Galipeau, 2020). This evidence has markedly shaped approaches to investigate and exploit alternative mechanisms that may be employed by MSCs to mediate therapeutic effects. We now recognize that the effects of MSCs following infusion could be largely paracrine mediated, and not by direct cell–cell contact (Caplan and Dennis, 2006; Caplan and Correa, 2011).

Soluble mediators may play more than one role in resolving tissue damage or pathology. These signals can be released directly into extracellular spaces to incite local responses or packaged in extracellular vesicles, e.g., exosomes, which travel to distal sites for systemic responses. The constituents of this cellular crosstalk described here are, in many cases, likely produced by MSCs that then directly influence the metabolic program and, in turn, the physiological function of TMφ. Alterations in macrophages are detected as skewed phenotypes as a result of metabolic reprogramming, although the duration of these temporal changes, downstream targets, and overall *in vivo* effects during pathology remain to be fully elucidated locally and systemically.

### COX/PGE2/EP4 Axis

**FIGURE 3:**
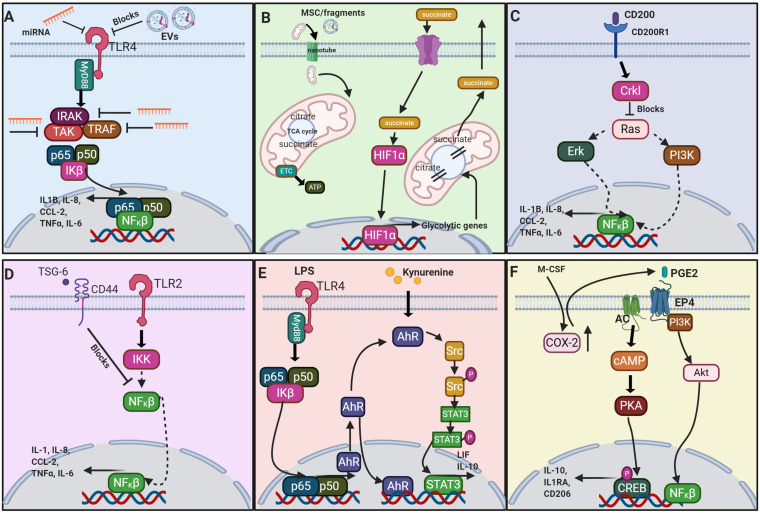
Schematic of a selection of the proposed signaling pathways which result from MSC or MSC derived factors interacting with macrophages. **(A)** The signaling of extracellular vesicles (EV) via negative regulation of the TLR4 (toll-like receptor 4) (Abdi et al., 2018); **(B)** metabolite signaling via the TCA cycle (Viola et al., 2019); **(C)** CD200-CD200R1 interaction (Manich et al., 2019); **(D)** TSG-6 via negative regulation of TLR2 (Choi et al., 2011); **(E)** AhR via TLR4 signaling (Hinden et al., 2015; Zhu et al., 2018); and **(F)** PGE2 (Na et al., 2015; Xu et al., 2017). miRNA, microRNA; TLR4, toll-like receptor 4; EV, extracellular vesicle; MyD88, myeloid differentiation primary response 88; IRAK, interleukin-1 receptor-associated kinase; TAK, TGF-β activated kinase; TRAF, TNF receptor-associated factor; p65, NF-κβ p65 subunit; p50, NF-κβ p50 subunit; I_K_β, NF_K_β inhibitor; NF_K_β, nuclear factor kappa light chain enhancer of activated B cells; IL1β, interleukin-1 β; IL-8, interleukin-8; CCL-2, C-C motif chemokine ligand 2; TNFα, tumor necrosis factor α; IL-6, interleukin-6; MSC, mesenchymal stromal cell; HIF1α, hypoxia-inducible factor 1-α; TCA, tricarboxylic acid cycle; ETC, electron transport chain; ATP, adenosine triphosphate; CD200, cluster of differentiation 200; CD200R1, CD200 receptor 1; Crkl, Crk-like protein; Erk, extracellular signal regulated kinase; PI3K, phosphoinositide 3-kinase; TSG-6, TNFα-stimulated gene 6; CD44, cluster of differentiation 44; TLR2, toll-like receptor 2; IKK, inhibitor of nuclear factor NF-κβ kinase, LPS, lipopolysaccharide; AhR, aryl hydrocarbon receptor; Src, proto-oncogene c-Src; Src-P, phosphorylated Src; STAT3, signal transducer and activator of transcription 3; LIF, leukemia inhibitory factor; IL-10, interleukin-10; M-CSF, macrophage colony-stimulating factor; COX-2, cyclooxygenase 2; PGE2, prostaglandin E_2_; EP4, E-type prostanoid receptor 4; AC, adenylate cyclase; Akt, protein kinase B; cAMP, cyclic adenosine monophosphate; PKA, protein kinase A; CREB, cAMP response element binding protein; IL-1RA, interleukin-1 receptor antagonist; CD206 (mannose receptor). Image created with BioRender.

One of the most well-known soluble mediators that has been attributed to the therapeutic effects of MSCs is prostaglandin E2 (PGE2). PGE2 is a homeostatic factor derived from the metabolism of arachidonic acid by prostaglandin synthases and cyclooxygenases (constitutively active COX1 and inducible COX2) in both myeloid and stromal cells (Kalinski, 2012). Both human and mouse MSCs constitutively produce PGE2, and upon pro-inflammatory challenge with interferon-γ (IFNγ), tumor necrosis factor-α (TNFα) or interleukin (IL)-1β, induced elevation of PGE2 has been demonstrated (Noronha et al., 2019). PGE2 promotes the production of interleukin-10 (IL-10) from TMφ and has a synergistic effect with indoleamine 2,3-dioxygenase (IDO) to elicit MSC-induced immunosuppression on various immune cells *in vitro* (Spaggiari et al., 2008; Nemeth et al., 2009). An MSC-dependent PGE2 has been demonstrated to alter monocyte-to-macrophage differentiation, promoting the survival of monocytes activated by macrophage colony stimulating factor (M-CSF), by *trans*-activation of the M-CSF receptor (Digiacomo et al., 2015) and, more importantly, polarized to an alternative activation (M2-like) phenotype (increased CD163 and CD206 and reduced MHCII/HLA-DR expressions) of TMφ (Figure 3F). Alternative activation macrophages upregulated secretion of amphiregulin (AREG) (Ko et al., 2020), showed bolstered functions of scavenging and phagocytic activities and enhanced production of immunomodulatory cytokines IL-10 and transforming growth factor-β (TGFβ) *in vitro* (Chiossone et al., 2016). MSCs from multiple tissues have reproducibly polarized macrophages of various sources by this PGE2-dependent mechanism resulting in the suppression of pro-inflammatory factors, e.g., tumor necrosis factor α (TNFα), IL-12p70, and IL-17, while promoting anti-inflammatory IL-10, ultimately inhibiting perpetuation of immune responses by antigen presentation (Melief et al., 2013; Deng et al., 2016; Manferdini et al., 2017). PGE2 binding to EP4 activates adenylate cyclase and intracellular cAMP levels are elevated. This in turn activates PKA and this has been shown to phosphorylate CREB (cyclic AMP-responsive element binding). Phosphorylated CREB leads to transcription of C/EBP-β which promotes anti-inflammatory gene expression (Na et al., 2015). Of all the PGE2 receptors, only EP4 was found to facilitate the production of IL-10 and suppression of TNFα (Yasui et al., 2015). Furthermore, the secreted molecules released by the M2-like macrophages induced regulatory T cells (Tregs), a T cell subset essential for immune tolerance (Schmidt et al., 2016).

### Tregs and AREG

MSCs skew TMφ phenotype via the secretome, with the resulting pro-regenerative macrophages demonstrating heightened release of IL10 and TGFβ (Francois et al., 2012; Mittal et al., 2016; Vasandan et al., 2016). However, MSC-conditioned macrophages are known to influence T cell activation *in vitro*, by inducing the differentiation of FoxP3^+^ Tregs from CD4+ helper T cells (Schmidt et al., 2016). Tregs are mediators of self-tolerance, essential to prevention of autoimmunity, and are immunosuppressive of inflammatory and allergic responses to infection. Tregs are recognized as significant contributors to immunomodulatory responses mediated by MSCs and in-depth descriptions of these interactions are described by Burr et al. (2013) and English (2013). MSC-primed macrophages, utilize TGFβ, IL-10 and other immunomodulatory factors that alter the differentiation of T cell subsets, dendritic cells and B cells *in vitro* as well as new *in vitro* and *in vivo* evidence points to epidermal growth factor receptor ligand AREG as another signaling moiety (Ko et al., 2020).

AREG has been implicated in the resolution of inflammation, regeneration of tissues, and restoration of homeostasis after injury. There are several ways that MSCs can promote the secretion of AREG from macrophages. These include the uptake of mitochondria from MSCs and the use of the COX-2/PGE2/EP4 signaling axis (Ko et al., 2020). In a mouse model of retinal inflammation, AREG suppressed immune responses by upregulating Tregs and downregulating Th1 cells. Recombinant AREG, administered alongside MSCs in macrophage depleted mice, showed some level of recovery of retinal pathology (Ko et al., 2020).

### Metabolic Reprogramming of TMφ by MSCs

The metabolic profile of the macrophage could be a key determinant of phenotype and function. Steady state macrophages exhibit a metabolism that utilizes glucose and oxygen for mitochondrial oxidative phosphorylation to generate energy in the form of ATP. Stimulation toward the classical activation phenotype *in vitro* demonstrated that a metabolic switch to glycolysis facilitated pro-inflammatory functions *in vitro* (El Kasmi and Stenmark, 2015). Classical activation (M1-like) macrophages stimulated by lipopolysaccharide treatment *in vitro*, indicated an upregulation of 31 metabolic enzyme/transporter-related genes which confirmed increased glycolysis, the citric acid cycle intermediate succinate, and release of pro-inflammatory IL-1β. Succinate was inferred as a key metabolite that enhances pro-inflammatory signaling during inflammation (Tannahill et al., 2013). This switch can be mitigated by the anti-inflammatory cytokine IL-10 (El Kasmi and Stenmark, 2015), suggesting multiple ways that MSCs modulate macrophage metabolism and thus phenotypic function. The reliance of M1 macrophages on glycolysis and the pentose phosphate pathway (PPP) appears to be related to two interruptions in the tricarboxylic acid (TCA)/Krebs cycle, which cause accumulation and exit of itaconate, succinate and citrate from the cycle. These metabolites are released from mitochondria, which both limits coupling of TCA to the electron transport chain (ETC) and also renders them capable of regulating cell metabolism. Succinate can stabilize HIF1α (Figure 3B) and thereby activate transcription of glycolytic genes, such that glycolysis is favored. Conversely, M2 macrophages appear to have an intact TCA cycle and so ROS are kept low and metabolites are not released to the cytoplasm (Viola et al., 2019). Upon receiving mitochondria from MSCs, M1-like (classical activation) macrophages polarized to M2-like (alternative activation) which resulted in a switch from glycolysis to oxidative phosphorylation. Therefore the exposure of MSCs to macrophages, and subsequent polarization to M2, is concomitant with a lower bioenergetic state with emphasis on catabolic pathways *in vitro* (El Kasmi and Stenmark, 2015; Vasandan et al., 2016). These catabolic pathways, involving β-oxidation of fatty acids, enhanced activity of 5′ AMP-activated kinase and reduced mTOR phosphorylation, are thought to rescue the macrophage from low tryptophan levels but have the obvious advantage of switching to energy conservation as well as a pro-regenerative TMφ state *in vitro* and *in vivo* (Phinney et al., 2015; Vasandan et al., 2016). During pathology, neural stem cells scavenge extracellular succinate, thwarting its utility by macrophages, in order to reduce infiltration of mononuclear phagocytes in neuroinflammation (Peruzzotti-Jametti et al., 2018). MSCs releasing insulin-like growth factor-2 under hypoxic conditions reprogram maturing macrophages to OXPHOS metabolism to improve neuroinflammation in a mouse model of Multiple Sclerosis (Du et al., 2019). These studies allude to targeting the metabolic programs of macrophages to regulate or restore the homeostatic balance of the milieu as potential therapeutic approaches.

Macrophages are in large part responsible for the development of atherosclerotic plaques since their activation by IFNγ produces foam cells, which will proceed to form unstable lesions in the intima of arteries. The elevated expression of scavenger receptors and CD36 on foam cells allows for increased uptake of low density lipoprotein (LDL) (Murray and Wynn, 2011) and release of cytokines locally that influence atherosclerosis pathology *in vivo* (Tedgui and Mallat, 2006). Other macrophage phenotypes have been identified in the plaque and can be atheroprotective (i.e., Mhem) (Boyle et al., 2009), pro-atherogenic (i.e., Mox) (Kadl et al., 2010) or both (i.e., M4) (Erbel et al., 2015). In an atherosclerotic mouse model, the use of skin-derived or human amnion-derived MSCs decreased plaque size in the arteries *in vivo* (Li et al., 2015; Wei et al., 2019). MSCs are implicated in reducing the aggregation of TMφ in the arterial intima (Shoji et al., 2011), inhibiting the formation of foam cells by elevating the number and function of Tregs *in vivo* (Wang et al., 2015) and by decreasing TNFα release (Li et al., 2015). All of these steps require regulation of TMφ polarization and modulation of the phenotypes in the plaque (Yang et al., 2020). In this way, MSCs sense the inflammatory environment and attempt to mitigate TMφ inflammatory responses.

### Oxidative Stress

MSCs counter oxidative insult by expressing antioxidant enzymes and heat shock proteins and upregulating redox-sensitive factors, such that lipid peroxidation and hydrogen peroxide (H_2_O_2_) and superoxide (${\text{O}}_{2}^{\cdot -}$) radical species are decreased *in vitro* (Oh et al., 2014). If free radical quenching, antioxidant production, switching of TMφ bioenergetics and mitochondrial transfer is all viewed as management of oxidative stress then this constitutes a significant feature of the interaction of MSCs with TMφ at sites of inflammation. Stanniocalcin-1, secreted by MSCs, decreases reactive oxygen species (ROS) generation, including mitochondrial ROS and suppresses the activation of the nucleotide-binding domain and leucine-rich repeat pyrin 3 (NLRP3) inflammasome *in vitro* (Oh et al., 2014). The NLRP3 inflammasome in activated macrophages senses damage-associated molecular patterns (DAMPS) and generates IL-1β to initiate the inflammatory cascade. Its activity can be quenched by co-culture with umbilical cord blood MSCs (Shin et al., 2016). Furthermore, when these MSCs were incubated with recombinant human IL-1β, their COX-2 expression was upregulated, and this suggests the idea of a feedback loop between the IL-1β from the inflammasome and MSC immunosuppression. In this case direct COX-2/PGE2 signaling is responsible for immunosuppressive effects on the inflammasome, in the absence of NO and IDO effects (Shin et al., 2016).

Reactive oxygen species also play a role in macrophage polarization in the heart. Resident cardiac TMφ are thought to be lost with age or after myocardial infarction (MI) and replacement may be inferior, due to lack of resident cells to engage in proliferation or the pro-inflammatory activity of monocytes recruited from the bone marrow (Weinberger and Schulz, 2015). In an MI study in mice, less apoptotic cardiomyocytes were observed in the infarct zone, after MSC infusion, and although both M2 and M1 macrophage levels were decreased, M2 was proportionately increased in the heart but not bone marrow *in vivo* (Dayan et al., 2011). Rat and mouse models of MI, with MSC administration, observed increases in alternative activation (M2-like) TMφ at the transplant site *in vivo* (Ben-Mordechai et al., 2013; Ishikane et al., 2013). CD146^+^ MSCs performed better than MSCs alone in a model of myocardial regeneration and this was attributed to a reduction in reactive oxygen species by the expression of CD146, an integral perivascular marker (Zhang et al., 2019a). In a follow up study, the total number of macrophages in the hearts of the mice did not vary after MSC transplantation but the ratio of M2:M1 increased (Zhang et al., 2019b). Injection of TNFα alongside the MSC transplantation abrogated the reparative effects of the MSCs *in vivo*. Although much is still unknown about macrophage subsets in the heart, MSCs appear to drive regeneration by reprogramming TMφ phenotypes.

### Message in a Bottle – MSC-Derived Extracellular Vesicles

The paracrine-mediated immunomodulatory factors secreted by MSCs are not solely attributed to the release of soluble molecules that act directly on cells in the local environment but also by uptake of those packaged in extracellular vesicles (EVs). MSC-derived EVs (MSC-EVs) are implicated as a cell-free product with, in some cases, comparable effects to infused MSCs, thus rendering MSC-EVs attractive candidates as therapy. Cultured MSCs secrete EVs that can be collected and subsequently isolated from the conditioned media (CM), enabling studies to perform comparisons using MSC-CM and MSC-EVs to determine the direct influences of MSC-EVs. Together, the ability to harness the therapeutic effects of MSCs without the need to deliver the cells make MSC-EVs attractive treatment alternatives under growing investigations in pre-clinical and clinical trials (Harrell et al., 2019a).

Apoptotic bodies (>1,000 nm in diameter) are the largest of the EVs which bud from MSCs during apoptosis. Microvesicles (MVs) are typically 100-1,000 nm in diameter and bud from the plasma membrane, and exosomes are the smallest EVs with diameters measuring 30–200 nm and result from budding of the late endosome membranes (Harrell et al., 2019b). The cargo packaged into MSC-EVs include many of the soluble cytokines and molecules discussed herein as well as other proteins, enzymes, organelles, lipids, metabolites, nucleic acids, and non-coding RNAs, all of which are comprehensively discussed in the context of inflammatory disease by Harrell et al. (2019b). The cargo that relays the “messages” to distant sites is still elusive yet is suggested to be highly specific (Baek et al., 2019).

MSC-derived exosomes are capable of inducing macrophage polarization to the alternative activation phenotype by several proposed mechanisms. MSC-derived exosomes have been attributed to macrophage polarization to the alternative activation phenotype with increased production and secretion of AREG by the described PGE2-dependent mechanism *in vitro* (Ko et al., 2020), suppression of the infiltration of classical activation macrophages and associated pro-inflammatory signaling (Mao et al., 2017; Willis et al., 2018a), and improved histoarchitecture by TMφ remodeling *in vivo* (Willis et al., 2018a) demonstrating alternative immunomodulatory delivery modalities that too can be supplied by MSCs (Figure 3A). Although the MSC-derived exosome-mediated effects are the result of the comprehensive mediators contained, depicting specific microRNA (miRNA) (Essandoh et al., 2016), cytokines, metabolites, and other molecules (Willis et al., 2018b) of which the EVs are comprised, may serve to identify other targeted therapeutics.

MSC-derived exosomes have been widely investigated in a number of pre-clinical studies of inflammatory diseases. TMφ induced to a classical activation phenotype and pro-inflammatory function that perpetuates inflammatory signaling were altered by MSC-derived exosomes to the alternative activation phenotype, resulting in attenuation of pathological severity in lung injury (Morrison et al., 2017; Wang et al., 2020), colitis (Mao et al., 2017), cardiomyopathy (Sun et al., 2018), retinal damage (Yu et al., 2016a), musculoskeletal conditions (Zhang et al., 2016; Cosenza et al., 2017), chronic wounds (Lo Sicco et al., 2017) and spinal cord injury (Lankford et al., 2018). MSC-derived exosomes not only mediated cellular improvements through TMφ, but also promoted the survival (Sun et al., 2018) and cytoprotection (Cosenza et al., 2017) of other vital tissue-specific cells *in vivo*. The mechanisms of action for MSC-derived exosomes remain to be fully elucidated, as multiple mediators are involved, and thus presumably more than one mechanistic effect, exerting comprehensive benefits during pathology.

### Organelle Donation and Bioenergetics

Organelles such as mitochondria too can be shuttled from MSCs to TMφ to support higher demands of macrophage physiological functions. For example, stimulation of TMφ phagocytosis in acute respiratory distress syndrome (ARDS) was elicited via nanotube transfer of MSC mitochondria to macrophages *in vivo* (Jackson et al., 2016). In addition, MSCs under intracellular oxidative stress *in vitro* will shuttle damaged mitochondria (containing an excess of oxidized and nitrosylated proteins) into microvesicles for extrusion to improve their bioenergetics *in vitro*. Simultaneous de-sensitization via miRNA-containing exosomes from the MSCs mitigates the activation of TMφ, which permits phagocytosis and re-use of the donated mitochondria by TMφ, a mechanism that promotes the survival of MSCs by outsourcing mitophagy (Phinney et al., 2015). Macrophages co-cultured with MSCs have shown enhanced phagocytic activity and this may be orchestrated by nanotube/EV-mediated mitochondrial transfer from MSCs (Ibrahim et al., 2014; Phinney et al., 2015; Ko et al., 2020). Although more evidence is necessary to determine the advantage of using MSC-EVs over MSCs, alternative strategies to elicit the comprehensive effects from MSC-macrophage crosstalk are promising.

## Contact-Dependent Communication

The complexities of the intercommunication between MSCs and TMφ may never be completely teased apart, and therefore we should expect possibilities of both indirect and direct mechanisms working in tandem to promote improvements to a pathological milieu. Moreover, the spatiotemporal microenvironment will continue to be altered to resolve inflammation and restore homeostasis, necessitating multiple functions of both the macrophages and MSCs. The dynamic heterogeneity of functions of both cell types are no coincidence; the integral crosstalk is the forefront to not only resolving pathology but improving our understanding of prophylactic measures to prevent disease. The observations of a number of *in vivo* investigations are presented in Table 2.

**TABLE 2:** A selection of *in vivo* studies using therapeutic MSCs with described pathology, source of MSCs, delivery route and outcomes.

Pathology	MSC source	Delivery route	Results/mediators observed	Citation
Acute lung injury	mBM-MSC	Intratracheal	↓neutrophils, NOS2, ↑Ym1, Arg1	Ionescu et al., 2012
Allergan-induced inflammation	mBM-MSC	IV	↓total cell count, IL-4, IL-13,IL-17 in BAL, ↓eosinophils, neutrophils in airway, ↑MSC in lungs	Xu et al., 2015
Asthma	mBM-MSC	IV	↑MSC in airways, *ex vivo ↓IL-6, IL-1β, NOS2*	Cui et al., 2020
Atherosclerosis	mBM-MSC	IV	↑Tregs, TGFβ, IL-10	Wang et al., 2015
Cardiomyopathy	mMSC-Exos	IV	↓apoptosis cardiomyocytes ↓M1, IL-1, IL-6, TNFα	Sun et al., 2018
Colitis	hUC MSC-Exos	IV	↓TNFα, IL-1β, IL-6, ↑IL-10	Mao et al., 2017
Colitis	canine ADMSCs	IP	↑TSG-6, IL-10, M2 ↓TNFα, IL-6,	Song et al., 2018
Collagen-induced arthritis	mBM-MSC	IV	↓serum TNFα, IL-1β	Luz-Crawford et al., 2016
Corneal allotransplantation	hBM-MSC	Peritransplant IV	↓IL-6, IL-1β, IL-12, ↓actn APCs, ↑TSG-6	Oh et al., 2012
Corneal injury	mBM-MSC	IV	↓IL-1β, inflam cell infiltration	Amouzegar et al., 2017
Cutaneous wound	hUC-MSC-Exo	Injected into wound	↓inflam cell infiltration, TLR4, p-P65, M1	Ti et al., 2015
Dermal injury	mBM-MSC	IV	↑transdifferentiation to skin cells	Sasaki et al., 2008
Dust mite asthma	mBM-MSC	IV	↑M2, IL-10, TGFβ1, ↓IL-6, MSCs only in M2s.	Braza et al., 2016
*E. coli pneumonia*	hBM-MSC	IV/intranasal	↓MIP-1α, MIP-1β, IL-27, IL-6, TNFα, ↑phagocytosis	Jackson et al., 2016
Experimental autoimmune encephalomyelitis	hUCMSC	IV	↓IL-1β, IFNγ, IL-17, ↑PD-L1, IGF-2	Du et al., 2019
GvHD	hBM-MSC	IP	↓GvHD effector T cells	Galleu et al., 2017
Intracerebral hemorrhage	rBM-MSC	IV	↓apoptosis, TMΦ, neutrophils, iNOS, MMP-9	Chen et al., 2015
Ischemia-reperfusion injury	Cardiac MSC	Intracardiac injection	↑CCR2+, CXCR1 + TMΦ,	Vagnozzi et al., 2020
Kidney ischemia-reperfusion	mBM-MSC	IV	↑MCP-1, MIP1α, IL-1β, 1L-10, TGFβ	Luk et al., 2016
LPS-induced abortion	mBM-MSC	IP	↓TNFα, IFNγ, IL-1β, IL-27, IL-6, ↑TSG-6	Li et al., 2019
Lung injury	mBM-MSC	IV	↓TNFα, IL1-RA	Ortiz et al., 2007
Lung injury	hBM-MSC	IV	↓neutrophils, ↑IL10, KGF	Devaney et al., 2015
Lung injury	hADMSC-Exo	IV, intratracheal	↓neutrophils in BAL, NFκβ, ↑IL-10, Arg1, miR-27a-3p in alveolar TMΦ	Wang et al., 2020
Myocardial infarction	hBM-MSC/hUC-MSC	IV	↓IL-1β, IL-6, apop cardiomyocytes, ↑IL-10, CD206+	Dayan et al., 2011
Osteoarthritis	hBM-MSC-CM	Intra-articular	↓MMP-13/TIMP-1, ↑autophagy chondrocytes	Chen et al., 2019
Retinal inflammation	hBM-MSC	IV	↓CD3+ cells ↑Treg	Ko et al., 2020
Retinal injury	mADMSC-Exos	Intravitreal	↓apoptosis, MCP-1, MΨ infiltration	Yu et al., 2016a
Sepsis	mBM-MSC	IV	↓TNFα, IL-6, ↑IL-10	Nemeth et al., 2009
Sepsis	Apoptotic rADMSC	IP	↓TNFα, MMP-9, NFκβ	Chang et al., 2012
Spinal cord injury	rMSC-Exos	IV	Exos in CD206 + Mψ only	Lankford et al., 2018
Spinal cord injury	hBM-MSC	Injected into injury	↑IL-4, IL-13, M2 TMΦ, ↓TNFα, IL-6, M1 TMΦ	Nakajima et al., 2012
Spinal cord injury	Dental pulp MSC-CM	Injected into injury	↑TGFβ, VEGF, CD206+, MCP-1, ED-Siglec 9,	Matsubara et al., 2015
Traumatic brain injury	hBM-MSC	IV	↑TIMP-3, ↓VEGF-A, blood brain barrier permeability	Menge et al., 2012

### Phagocytosis of Dead/Apoptotic Cells

Mechanisms involved in recognition of apoptotic cells and the consequential removal by phagocytosis, involve receptors classed as C-type lectin, vitronectin (with assistance from CD36 and thrombospondin on apoptotic cells), phosphatidylserine, scavenger proteins (bind apoptotic, necrotic cells, opsonized pathogens and cell debris), TLRs and macrophage antigens. Phagocytosis of apoptotic cells has been shown to inhibit macrophage production of a range of cytokines, except TGFβ1, PGE2 and platelet activating factor (PAF) *in vitro* (Fadok et al., 1998). In general, the result of phagocytosis appears to be immunosuppression. Recently, a novel cell contact-dependent mechanism demonstrated a lipoprotein receptor protein mediated uptake of MSC-derived cytoplasmic components, or processing bodies, by monocytes and macrophages resulted in reprogramming to an anti-inflammatory function. Reprogrammed monocytes and macrophages were able to significantly suppress activated helper T cells proliferation *in vitro* and mitigate inflammation in a small animal model of LPS-induced lung inflammation (Min et al., 2020). In a pre-clinical model of asthma, polarization of alveolar TMφ was accompanied by phagocytosis of PKH26 + MSC and upregulation of TGFβ and IL-10 mRNA (Braza et al., 2016) and M2 markers *ex vivo* were only expressed on macrophages which had ingested MSCs.

Indeed the daily clearance of fetal apoptotic stromal cells by maternal resident lung macrophages, stimulating IL-10 release and IL-1β suppression, facilitates immunotolerance *in vivo* (Abumaree et al., 2006; Galipeau and Sensebe, 2018). Efferocytosis, a term originally coined in 2003, is the targeted removal of apoptotic cells, as well as cells dying through the many other forms of cell death (Henson, 2017). Phagocytosing macrophages clear apoptotic MSCs, and this process precipitates intracellular signaling in macrophages to downregulate TNFα and NO production, in favor of TGFβ1 and IL-10 *in vitro* (Braza et al., 2016; de Witte et al., 2018). Therefore, MSCs may take part in immunoregulation and macrophage polarization even after becoming apoptotic.

These studies allude to the state of MSCs when delivered *in vivo*. Freshly thawed cells, in general, have a higher metabolic activity, greater percentage of apoptotic cells and a higher necrotic fraction than culture rescued cells (Antebi et al., 2019). MSCs used within 24 h of thawing, show compromised T cell suppression, increased susceptibility to lysis by complement or immune cells and shortened persistence *in vivo* with intravenous (IV) administration (Moll et al., 2016). It has also been shown that roughly 50% of IV transfused mouse MSCs become trapped in the lung and are ultimately phagocytosed by lung resident macrophages (Nemeth et al., 2009). In an ischemia-reperfusion injury model in mice, cardiac MSC injection improved heart function, not by production of new cardiomyocytes but by induction of CCR2^+^ and CX3CR1^+^ TMφ. Changes in the local extracellular matrix content of the peri-infarct border zone *in vivo* occurred whether the MSCs were live or freeze-thawed (non-viable) and could be substituted by a chemical inducer of the innate immune response (zymosan) (Vagnozzi et al., 2020). Therefore, this may be an indirect effect of the MSCs and not paracrine-mediated, but nevertheless, MSCs whether viable, intact or even apoptotic, cell signaling still produces a beneficial effect on the resulting macrophage functional output.

It is therefore possible that MSCs can be apoptotic, metabolically inactivated or fragmented even (membrane particles) and still exert immunomodulation (Luk et al., 2016; Goncalves et al., 2017). Apoptotic adipose-derived MSCs were able to improve survival of rats in a model of sepsis by decreasing TNFα levels in circulations as well as the frequencies of systemic and splenic helper and cytotoxic T cells (Chang et al., 2012). Evidence supports that apoptotic cells were more potent than viable cells in lung, kidney injury and ischemia-reperfusion models (Sung et al., 2013). Furthermore, the deliberate perforin-mediated induction of apoptosis of MSCs by cytotoxic cells in a murine model of GvHD, appeared necessary for the success of the MSC infusion (Galleu et al., 2017). Induction of caspase 8 and apoptosis was necessary for immunosuppression *in vivo* and patients with high cytoxicity were more likely to respond to MSCs, indicating a bifurcation of patient responses. Intraperitoneally (IP)-delivered MSCs were sequestered in the phagocytes in lymph nodes and IV-delivered MSCs homed to the lungs. These apoptotic MSCs only improved GvHD outcomes when delivered IP, and not IV, and no IDO was induced by IV-administered MSCs. Regulation of pro-inflammatory Th1 and Th17 cells by MSC-derived PGE2 only occurred in the presence of CD14^+^ cells in PBMC cultures, therefore indicating a reliance on myeloid cells for immunoregulatory mechanisms (Rozenberg et al., 2016).

The heat inactivation (HI) protocol of Luk et al. (2016) introduced in 2016 incubated MSCs for 30 min at 50°C (HI MSCs) and this resulted in the lack of a secretory profile, no proliferative or metabolic activity and a disintegration of the cell without heat shock protein release. HI MSCs did not inhibit T cell proliferation but were able to reduce TNFα release by monocytes challenged with LPS *in vitro* (Luk et al., 2016). This points to a non-specific immunosuppression, i.e., independent of cell viability, by the reticuloendothelial system of the host (Poon et al., 2014). Indeed, studies in our laboratory, using MSC suppression of TNFα release by THP-1 macrophages, have found both freshly thawed and cultured MSCs able to exert immunosuppressive effects and yet the culture-rescued cells generally have few apoptotic and necrotic cells post harvest, as mentioned earlier (Pradhan P et al. BioRxiv doi: https://doi.org/10.1101/2020.09.12.294850).

### Aryl Hydrocarbon Receptor on MSCs

Aside from the secretome and engulfment of MSCs, a proposed mechanism mediated by MSCs resulting in macrophage polarization, or macrophage phenotype “plasticity,” is by the activation of the aryl hydrocarbon receptor (AhR). This MSC receptor responds to environmental stimuli and contributes to both physiological cell development and immune regulation (Abney and Galipeau, 2020). The AhR, when bound by ligands of environmental pollutants, translocates from the cytoplasm to the nucleus and facilitates AhR-related transcription of genes, which typically elicit immunotoxicological effects. For example, MSCs upregulate cytochrome P450 isoforms, *cyp1a1* and *cyp1b2* genes in response to cockroach allergen *in vitro* (Xu et al., 2015). This AhR receptor activation is kynurenine-mediated and results in immunosuppressive alterations [decreased IL-6 expression and enhanced leukemia inhibitory factor (LIF) *ex vivo* (Hinden et al., 2015)] (Figure 3E). This suggests an immunomodulatory potential of MSC directly regulated by AhR.

In mice treated with MSCs prior to intratracheal cockroach extract (CRE) challenge, there was a significant decrease in bronchial inflammation and goblet cell hyperplasia. Isolated lung TMφ showed a significant increase in alternative activation (M2-like) marker expressions (e.g., Arg1, FIZZ1, and Ym1) relative to CRE treatment alone, suggesting treatment with MSCs polarized macrophages to an alternative activation phenotype under allergen-induced pulmonary inflammation *in vivo* (Cui et al., 2020). The IDO inhibitor 1-methyl tryptophan can activate AhR in MSCs *in vitro* (Lewis et al., 2017) and the AhR-Src-STAT3-IL10 signaling pathway has been pivotal to controlling inflammatory macrophages *in vitro* (Zhu et al., 2018). The correlation between this signaling pathway and immunomodulatory mechanisms by MSCs may be a key axis for targeted approaches.

### CD200, TSG-6, and Hormone Receptors

Another contact-dependent interaction implicated in macrophage polarization is the CD200/CD200R1 receptor complex. CD200 (OX-2) is a transmembrane glycoprotein and its counterpart, CD200-R1, is found on myeloid cells and T cells. Interestingly, the role of CD200 cannot be fully extricated from soluble tumor necrosis factor stimulated gene-6 (TSG-6) signaling. In an LPS-induced abortion mouse model, TSG-6-silenced or CD200-silenced MSCs exhibited a higher embryo resorption rate and both had higher levels of TNFα, IFNγ, and induced nitric oxide synthase (iNOS) in the decidua than non-silenced control MSCs supporting the CD200- (cell-mediated) and TSG-6-dependent (i.e., paracrine-mediated) mechanism (Li et al., 2019) (Figure 3C). This evidence gives credence to the idea that MSCs exert immune tolerance by both cell-contact as well as paracrine-mediated mechanisms.

The ability of bone marrow MSCs to suppress TNFα release by IFNγ-primed THP-1 macrophages appeared correlated to the levels of CD200 (Pietila et al., 2012). High expression of CD200 on umbilical cord derived MSCs was associated with improved immunomodulatory effects *in vitro* and lack of CD200 expression was correlated to poor suppressive capacity of the MSCs, suggesting a link between CD200 expression on MSCs and suppression of pro-inflammatory macrophage signaling (Pietila et al., 2012). TSG-6 released by MSCs is a signaling molecule that has been the focus of several pathological conditions. TSG-6 deletion in MSCs abrogated the ability to repair corneal damage, myocardial infarct and aid in corneal allograft survival (Oh et al., 2012). The role of TSG-6 signaling has been explored in inflammatory bowel disease (IBD), where canine adipose tissue-derived MSCs induced polarization of TMφ in murine IBD, resulting in more M2 TMφ released into the colon and improvements in disease activity index (Song et al., 2018). TSG-6, released by the MSCs, prevented blood brain barrier (BBB) disruption in intracerebral hemorrhage in rats and reduced the density of microglia/macrophages at the hemorrhage site (Chen et al., 2015). TSG-6 has a known interaction with the CD44 receptor on TMφ, which blocks TLR2-mediated translocation of nuclear factor kappa κβ (NFκβ) to the nucleus alleviating inflammatory signaling *in vitro* (Choi et al., 2011).

In pathological conditions, MSCs have been shown to block the translocation of NFκβ to the nucleus, indicative of the TSG6/TLR2/NFκβ pathway. LPS and IFNγ trigger intracellular signaling pathways, via degradation of Iκβ, which frees NFκβ to translocate to the nucleus to bind promoters of pro-inflammatory mediators (Figure 3D). LPS-induced lung injury was lessened by treatment with MSCs or MSC-CM, with alveolar macrophages showing heightened Ym1 and decreased iNOS (NOS2) compared to untreated controls *ex vivo* (Ionescu et al., 2012). However, the signaling pathways of activated resident macrophages are complex and difficult to study *in vivo*, with considerable heterogeneity of response to different stimuli. The transcriptome of activated macrophages revealed nine distinct activation programs, a spectrum of activation much more advanced than the M1/M2 classification conventions (Xue et al., 2014).

Apart from modulation of TMφ phenotypes, MSCs also play a role in blocking the differentiation of steady state myeloid progenitors under inflammatory conditions and the subsequent infiltration of inflammatory effector cells at the site of inflammation. Under homeostatic conditions, bone marrow-derived MSCs support hematopoiesis, maintaining hematopoietic stem cells (HSCs) in an undifferentiated state via trophic factor release. Under inflammatory conditions (i.e., high levels of IFNγ, IL1β, and TNFα), however, HSCs undergo myelopoiesis, resulting in their differentiation to macrophages and neutrophils. CD200 expressed on MSCs is proposed to be responsible for the suppression of inflammation and the maintenance of myeloid progenitors in an undifferentiated state *in vitro* and *in vivo* (Amouzegar et al., 2017). In a mouse model of corneal injury, systemically administered control MSCs showed a fivefold induction of myeloid progenitors in the cornea and a concomitant reduction in inflammatory cells and IL-1β, compared to mice injected with (silenced) CD200-shRNA-treated MSCs (Amouzegar et al., 2017).

Besides PGE2-EP4, TSG-6-CD44, and CD200-CD200R1, signaling between progesterone receptors (PR) and glucocorticoid receptors (GR) on microglia, the macrophages of the CNS, has been implicated in the triggering of microglial polarization *in vivo* (Xu et al., 2017). Progesterone has been shown to be neuroprotective in pre-clinical models of traumatic brain injury, by inhibition of microglial activation and prevention of inflammatory cytokine release (Lopez-Rodriguez et al., 2015). Inhibition of PR and GR by mifepristone partly blocked human placental MSC-driven polarization of macrophages. In this study, the basal release of soluble factors by MSCs suggested TGFβ as a key mediator of the resulting immunomodulation (Abumaree et al., 2013).

MSC-macrophage interactions in bone (re)modeling appear to be paracrine- and contact-mediated via CD200/CD200R *in vitro* (Varin et al., 2013). Osteoclasts (bone resorbing cells) can differentiate from hematopoietic precursor cells or other macrophage lineage cells. Activated TMφ, which release pro-inflammatory cytokines, can disrupt the balance of osteoclast-mediated bone resorption and osteoblast-mediated bone formation, resulting in bone loss (Yang and Yang, 2019). However, depletion of TMφ during intramembranous bone deposition in fracture repair led to impaired healing (Alexander et al., 2011). Soluble CD200 can inhibit differentiation of osteoclast precursors and inhibit receptor activator of nuclear factor kappa-β ligand (RANKL) signaling. MSCs expressing CD200 can block osteoclast formation and resorption pit activity *in vitro* (Varin et al., 2013) and CD200R inhibition can result in hyperactivation of macrophages and increased susceptibility to autoimmune diseases (Wright et al., 2003). Future research should unravel the reliance on contact dependent vs. soluble mediators for bone regulation and pathology.

## MSCs Altered by Macrophages

Macrophage conditioned media, as well as co-culture with MSCs, can influence MSCs viability and secretome (Freytes et al., 2013). M2 macrophages are reported to produce more osteoactivin/gpnmb and thereby activate the ERK/JNK signaling pathway to assist MSC survival, proliferation and migration (Yu et al., 2016b; Xu et al., 2017). LPS-induced TNFα release by macrophages can stimulate MSCs to secrete growth factors that promote tissue repair (Crisostomo et al., 2008) and drive MSCs to release inflammatory cytokines (Abumaree et al., 2013).

It is notable that M1 macrophage-MSC co-cultures demonstrated markedly higher upregulated genes compared to than equivalent M2 macrophage-MSC co-cultures (Espagnolle et al., 2017), verifying that the macrophage program can specify gene expression and cell-mediated immune responses. Upregulated genes in M1-MSCs cultures included *IDO*, *COX2* (immunosuppressive genes), *PDL-1*, *CD54* (MSC and T lymphocytes), *CXCL9* and CXCL10 (involved in T cell trafficking). M1-primed MSCs showed stronger inhibition of T cell proliferation, likely through a homotypic CD54 synapse between M1 macrophage and MSC (Espagnolle et al., 2017).

Other evidence of the effects of macrophage-primed MSCs can be found in orthopedic research, and relate to the multi-tissue compartment of the joint. M2 macrophages, co-cultured with MSCs, drive the expression of alkaline phosphatase, osteogenic markers and bone mineralization to regenerate bone (Champagne et al., 2002) and the expression of chondrogenic and clonogenic genes, to aid cartilage formation (Sesia et al., 2015). Similarly, exosomes isolated from LPS-treated monocytes increased gene expression of Runx2 and BMP-2 in human MSCs upon exposure *in vitro* (Ekstrom et al., 2013). Synovial M1 macrophages promote upregulation of proteolytic enzymes in osteoarthritis and negatively impact MSC chondrogenic effects on chondroprogenitors (Fahy et al., 2014). These key investigations shed light on the potential MOAs of MSCs in musculoskeletal indications, however, the dark side of the molecular crosstalk between MSCs and TMφ reveals how these potential MOAs can be exploited during carcinogenesis.

Although this review is intended to focus on MSCs administered as therapy, several studies have also reported a central crosstalk between MSCs and TMφ in the context of cancer. Given that MSCs are defined as a cultured cell type and their *in vivo* identity prior to isolation still remains unclear [i.e., pericyte-like (Caplan, 2008)], explorations to reveal the influences of MSCs within the tumor microenvironment are limited. Often, culture expanded MSCs are co-cultured with cancer cells and TAMs isolated from tumors or injected directly into the *in vivo* tumor microenvironment to investigate the influences of MSCs. Furthermore, it is challenging to elucidate the phenotypes, functions, and crosstalk attributed to the various cells within the *in vivo* tumor microenvironment that may, collectively, promote or mitigate cancer progression.

Like TMφ, the paradigm of polarized responses resulting from TLR signaling has also been described for MSCs (i.e., MSC1 and MSC2), suggesting pro- or anti-inflammatory effects (Tomchuck et al., 2008; Waterman et al., 2010). Waterman et al. (2012) suggested that MSC1 attenuated tumor growth *in vitro* and *in vivo*, whereas MSC2 had the opposite effect of promoting tumor growth and metastasis, linking the secretory profiles of MSCs directly to alterations to cancer cells. The changing tumor microenvironment likely alters, “educates,” or even “hijacks” (Quail and Joyce, 2013) MSCs as well as tumor-associated macrophages (TAMs). For example, key findings have linked inflammation and cancer progression by elucidating the roles of polarized TAMs and their activation of MSCs. Anti-tumor effects have been attributed to M1-like TAMs and, in contrast, multiple aspects of tumor progression are correlated with the suppressive program of M2-like TAMs. Inflammation in the tumor microenvironment produced an M1 phenotype of TAMS which, in turn, induced an immunosuppressive profile of MSCs, expressing high levels of iNOS and MCP1. Further recruitment of TAMs mediated by MCP1 secreted from MSCs along with IL-6 led to polarization into an M2-like phenotype which promoted tumor growth (Jia et al., 2016). Our contextual understanding regarding the crosstalk between TAMs and endogenous cells during carcinogenesis is far from being fully understood. A greater appreciation of the crosstalk between MSCs and TAMs as well as the development of cancer stem cells in cancer research can be found in more focused reviews by Papaccio et al. (2017) and Ridge et al. (2017).

## Conclusion

Scientific understanding is continually enriched and reshaped by technological advancements, research methodologies and new discoveries. As our understanding of monocytes and macrophages has evolved recently, so has our viewpoint about MSCs. There is now broad agreement that MSCs are not in fact stem cells and likely do not exhibit multipotency when delivered *in vivo*; rather, they are potent signaling cells with great plasticity, that interact dynamically with their microenvironment, e.g., with TMφ, to modulate and control immune homeostasis and produce, or help produce, various pro-regenerative signals.

As with current assays that evaluate *in vitro* functional or therapeutic potencies of MSCs, a developed assay based on an identified MOA needs to account for many considerations that may obscure the reproducibility of results – highlighting the importance of standardization of all processes from harvest to delivery of MSCs. One must consider the biological variation of each donor and alterations imparted by different manufacturing processes including, how the cells were isolated, stored, shipped, cultured, expanded, and delivered (e.g., route, timing, and dose). A major realization is that MSCs are mainly administered by intravenous infusion, destined for entrapment in the lungs – a tissue that may be far from the site of pathology. Thus, the applicability of a given MOA with respect to the route of delivery and site of pathology for treatment must be considered, although the predominant therapeutic effects of MSCs could be via paracrine activities (Giri and Galipeau, 2020). Furthermore, we refer to therapeutic MSCs as a culture-based cell type confined by an identity characterized *ex vivo* that are then re-introduced to an *in vivo* milieu that is highly variable from patient to patient. This alludes to the difficulty in developing *in vitro* assays that are predictive of *in vivo* outcomes. The complexity of identifying and validating potential MOAs mediated by therapeutic MSCs bolster the need for deep and broad characterization of the cells especially using multi-omic analyses, better understanding of the critical process parameters that can help produce cells with consistent and reproducible quality, identification of the critical quality attributes that are predictive of the product quality and patient outcomes, standardization of processes and analytical methods, pertinent *in vitro* potency and safety assays, appropriate animal models for *in vivo* pre-clinical validation, and well-designed randomized controlled trials to evaluate clinical efficacy.

The evidences from pre-clinical studies, to date, suggest that MSC-macrophage crosstalk may play a critical role in their *in vivo* function and can be a potential MOA. These interactions are largely a result of the MSC secretome, including soluble factors, mitochondrial donation, mediating complex pathological milieus characterized by pro-inflammatory, metabolic, proliferative, differentiative, hypoxic, REDOX mediators, along with some cell contact-dependent mechanisms. Together, these studies shed light on the various modes in which MSCs alter macrophage phenotype and, in so doing, can modulate local and systemic immunopathology to promote repair and restore homeostasis.

As mentioned earlier, the majority of mechanisms by which macrophages and MSCs interact have been discovered *in vitro* and much more *in vivo* studies are needed to tie in these *ex vivo* observations to those occurring in the body upon administration. Through correlation of *in vitro* functional assays to qualitative and quantitative *in vivo* effects, we should be able to identify potency assays which are more representative of *in vivo* performance and employ these to inform the manufacturing of MSCs for mainstream clinical therapy. The potential for MSC therapeutics lies in the ability to improve our understanding of how we can best harness their key communication mechanisms with other cells, and reproducibly promote the beneficial effects, ultimately translating benchtop discoveries to bedside MOAs to advance these promising therapies into clinic and the industry.

## Author Contributions

HS and AB contributed to the preparation, writing, and review of the manuscript. CY and KR contributed to the writing and review of the manuscript. All authors approved the submitted version.

## Conflict of Interest

The authors declare that the research was conducted in the absence of any commercial or financial relationships that could be construed as a potential conflict of interest.
